# IL-22 ameliorates LPS-induced acute liver injury by autophagy activation through ATF4-ATG7 signaling

**DOI:** 10.1038/s41419-020-03176-4

**Published:** 2020-11-11

**Authors:** Lujing Shao, Xi Xiong, Yucai Zhang, Huijie Miao, Yuqian Ren, Xiaomeng Tang, Jia Song, Chunxia Wang

**Affiliations:** 1 Department of Critical Care Medicine, Shanghai Children’s Hospital, Shanghai Jiao Tong University, Shanghai, 200062 China; 2 Institute of Pediatric Critical Care, Shanghai Jiao Tong University, Shanghai, 200062 China

**Keywords:** Predictive markers, Experimental models of disease

## Abstract

Uncontrollable inflammatory response acts as a driver of sepsis-associated liver injury (SALI). IL-22 plays an important role in regulating inflammatory responses, but its role in SALI remains unknown. The aim of the study was to assess the association of serum IL-22 with SALI in pediatric patients and to enclose the underlying mechanisms of IL-22 involved in lipopolysaccharide (LPS) - induced acute liver injury (ALI) in mice. Serum IL-22 levels in patients with SALI were significantly lower than in septic patients without liver injury, and the area under receiver operating characteristic (ROC) curve of IL-22 for discriminating SALI was 0.765 (95% *CI*: 0.593–0.937). Pre-administration of recombinant murine IL-22 alleviated LPS-induced ALI in mice, and serum IL-6 levels and the mRNA levels of TNF-α, IL-1β, and IL-6 in livers were decreased in response to IL-22 pre-treatment in mice. More importantly, IL-22 pre-treatment activated hepatic autophagy mediated by activating transcription factor 4 (ATF4)-autophagy-related gene 7 (ATG7) signaling in vivo and in vitro in response to LPS administration. Moreover, knockdown of ATF4 in mice aggravated LPS-induced ALI, which was associated with suppressed ATG7-related autophagy. In addition, the protective effects of IL-22 on LPS-induced ALI was partially blocked by ATF4 knockdown, which was associated with lower expression of LC3II/I in the livers of ATF4 knockdown (HT or *Atf4*^+/−^) mice compared with wild-type mice (WT or *Atf4*^+/+^) mice. In conclusion, low serum IL-22 level is associated with SALI occurrence, and IL-22 pre-administration activates autophagy in hepatocytes and protects mice against LPS-induced ALI partially related to ATF4-ATG7 signaling pathway.

## Background

Sepsis is a life-threatening organ dysfunction caused by dysregulated immune response to infection^1^. Liver plays a key role in immune response and releases amounts of inflammatory factors, yet it is also vulnerable to inflammatory damage. Sepsis-associated liver injury (SALI), as a common clinical feature, is an independent risk factor for multiple organ dysfunction and sepsis-induced death^2^. Developing potential new therapies or optimizing the management is crucial for restoring liver function and improving mortality rates in patients with SALI.

Interleukin-22 (IL-22), as one member of IL-10 cytokine family produced by T-helper (Th)17, Th22, natural killer (NK) cells, is involved in inflammatory reaction^3,4^. Extensive evidences indicated that IL-22 plays a protective role in epithelial tissues regeneration in response to infection^5–7^. Especially, IL-22 is a master regulator for maintaining intestinal barrier integrity^6,8^. Usually, IL-22 binds to IL-10 receptor 2 (IL-10R2) and the IL-22 receptor 1 (IL-22R1) complex and then stimulates downstream signaling including signal transducer and activators of transcription 3 (STAT3), c-jun N-terminal kinase (JNK), and mitogen-activated protein kinase (MAPK) pathways^9^. Beside of gastrointestinal tract, IL-22R is widely expressed in liver, and IL-22 was proved to be involved in abdominal sepsis^3,10,11^. In livers, several studies indicated that IL-22 enhances AMP-activated protein kinase (AMPK)-dependent autophagy^12^ and induces production of acute phase protein, mitogenic proteins and anti-apoptotic proteins to exert a hepato-protective effect^13,14^. However, there are some studies indicated that IL-22 might contribute to pathophysiologic inflammation in liver damage. IL-22 exacerbates chronic liver inflammation and fibrosis by promoting Th17 cells recruitment in HBV-infected patients and HBV-transgenic mice^15^. In addition, IL-22 may contribute to the liver fibrogenesis under HCV infection via increasing the expression of α-smooth muscle actin (α-SMA) accompanied with inhibited cell apoptosis, promoted cell proliferation^16^. All these results have led to a conflicting conclusion to IL-22 regulating inflammation response in livers under acute or chronic inflammatory damage. According to previous evidences, IL-22 is widely reported as a survival factor of hepatocyte^13,14^. Until now, serum IL-22 levels in pediatric patients with SALI and the potential roles of IL-22 in SALI remain unclear. In the present study, we hypothesized that IL-22 could be a protective factor in patients with SALI.

In the current study, serum levels of IL-22 were determined in pediatric patients with sepsis, and the potential roles and underlying mechanisms of recombinant mouse IL-22 was investigated in lipopolysaccharide (LPS)-induced acute liver injury (ALI) and cells treated with LPS. Overall, our data demonstrated that serum levels of IL-22 are lower in patients with SALI than those without ALI. IL-22 pre-treatment activates autophagy in vivo and in vitro and alleviates LPS-induced ALI and hepatocyte apoptosis partially related to activating transcription factor 4 (ATF4)- autophagy-related gene 7 (ATG7) signaling pathway.

## Materials and methods

### Patients

Forty-one pediatric septic patients were enrolled who were admitted to pediatric intensive care unit (PICU) of Shanghai Children’s Hospital from January 2018 to December 2018. Pediatric sepsis was defined according to the clinical criteria for sepsis based on the International Pediatric Sepsis consensus conference in 2005^17^. SALI (total bilirubin ≥4 mg/dL or ALT 2 times the upper limit of normal for age), sepsis-associated respiratory failure (sepsis-RF) (PaO_2_/FiO_2_ < 300 mmHg in the absence of cyanotic heart disease or pre-existing lung disease), or sepsis-associated acute kidney injury (sepsis-AKI) (serum creatinine 2 times the upper limit of normal for age or 2-fold increase in baseline creatinine) was defined according to the criteria of International Pediatric Sepsis Consensus Conference^17^. Sepsis-associated gastrointestinal (GI) dysfunction (sepsis-GI dysfunction) (absent bowel sounds) was defined by Surviving Sepsis Campaign International Guidelines in 2012^18^. Patients were diagnosed with organ dysfunction after admission to PICU even throughout PICU stay. Patients with advanced tumor, hereditary metabolic diseases, and the use of immunosuppressive drugs in the past one month were excluded. The clinical data including Pediatric risk of mortality (PRISM) III score, the counts of white blood cells (WBC), C-reactive protein (CRP), alanine aminotransferase (ALT), aspartate aminotransferase (AST), serum IL-22 level on PICU admission, the length of PICU stay, and PICU mortality, were recorded.

This protocol was approved by the Clinical Research Ethics Committee of Shanghai Children’s Hospital affiliated to Shanghai Jiao Tong University (Approval number: 2018R039-F01), and the informed consent was signed by the patients’ relatives.

### Animal experiments

Male C57BL/6 mice (8-week old) were purchased from the Shanghai Model Organisms Center (Shanghai, China). The care and use of the mice were conducted according to a protocol that was approved by the Ethics committee of the Children’s Hospital Affiliated to Shanghai Jiao Tong University (Shanghai, China). ATF4 gene knockdown mice (HT or *Atf4*^*+/*−^) were purchased from Model Animal Research Center of Nanjing University. For determining the serum levels of IL-22 in response to LPS stimulation, mice were intraperitoneally injected LPS (5 mg/kg, *E. coli* 0111: B4, Sigma - Aldrich Co.) to induce ALI. The mice were randomized and euthanized at 0, 1, 2, 4, 8, 24, and 48 h after LPS treatment (*n* = 5). Peripheral blood samples were collected for further ALT, AST and interleukin-6 (IL-6) levels measurement and liver tissues were collected for further analysis.

To explore the function of IL-22 protecting mice against LPS-induced ALI, a total of 24 male mice (8-week old) were randomized and divided into four groups including control (Control), LPS treatment (LPS), only IL-22 treatment (IL-22), and pretreatment with IL-22 followed by LPS administration (IL22 + LPS). Recombinant murine IL-22 (PeproTech, London, U.K.) was intravenously injected in a dose of 100 μg/kg body weight for 4 days before LPS treatment. PBS was delivered in a similar fashion as control vehicle (*n* = 6 per group). The phenotype of LPS-induced ALI in *Atf4*^*+/*−^ or wild type (*Atf4*^*+/+*^) mice was compared. Furthermore, the protective effects of IL-22 on LPS-induced ALI were investigated in *Atf4*^*+/*−^ compared with *Atf4*^*+/+*^ mice. The mice were euthanized at 24 h after LPS treatment. Peripheral blood samples and liver tissues were collected for further analysis.

### Blood sampling and cytokine measurement

The blood samples were collected within 1 h of PICU admission in sterile vacutainer tubes for further analysis. Serum was obtained and stored at −80 °C for IL-22 determination. The concentration of human IL-22 was determined by human IL-22 ELISA kit (MultiScience [LIANKE] Biotech, CO., LTD, Hangzhou, China). Serum IL-22 and IL-6 levels in mice were determined with commercially available ELISA kits (MultiScience [LIANKE] Biotech, CO., LTD, Hangzhou, China) according to the manufacturer’s instructions. The levels of ALT and AST were analyzed in our clinical laboratory center. The technician was blinded to the group allocation during the experiment.

### Histopathology

Liver samples were harvested and fixed in 4% paraformaldehyde overnight at 4 °C, then embedded in paraffin and 4-μm thickness sections were mounted on glass. Tissue sections were stained with hematoxylin and eosin (*H&E*) and visualized under light microscopy. The technician was blinded to the group allocation during the experiment.

### Cell culture and treatments

HepG2 and Hepa1-6 cells were purchased from Cell Resource Center of Shanghai Academy of Biological Sciences, Chinese Academy of Sciences, and all cell lines were tested and proved to be negative for mycoplasma contamination. HepG2 and Hepa1-6 cells were cultured in DMEM with 10% FBS at 37 °C in a humidified incubator (5% CO_2_). LPS (1 μg/ml) was added into cell culture for 8 h. IL-22 was used to pre-treat cells in a dose of 10 ng/ml for 24 h, then, cells were treated with LPS for another 8 h. To investigate the effects of autophagy activation on IL-22-mediated protection, 3-methyladenine (3-MA) was used in a dose of 5 mM for 24 h before IL-22 treatment. To explore the role of ATF4 in IL-22 protecting cell apoptosis against LPS stimuli, cells were infected with lentivirus-mediated knockdown of ATF4 (Lentivirus-shATF4) system (MOI = 15) (siATF4: 5’-gaguuaguuugacagcuaatt*-*3’*)* for 24 h. All cell culture experiments were repeated for three times, and three duplicates per group each time.

### Cell counting

Cells were harvested using 0.25% trypsin, washed with phosphate-buffered saline (PBS). Then, cell counting was performed on the acquired images (Image-Pro Plus Version 6.2; Media Cybernetics Inc., MD, USA).

### Transmission electron microscopy

HepG2 Cells were pre-treated with or without IL-22 (10 ng/ml) for 24 h, then be treated with LPS (1 μg/ml) for 8 h. To investigate the change of autophagosome, cells were harvested and fixed at 4 °C overnight in 2.5% glutaraldehyde, postfixed at temperature in 1% osmium tetroxide for 2 h, then cells were embedded and obtained ultra-thin sections. The sections were stained with uranyl acetate/lead citrate and analysis by transmission electron microscopy (HITACHI HT7700) at 60 kV.

### Western immunoblotting

Liver samples and cells were homogenized in RIPA lysis buffer (Beyotime Biotechnology). Samples were resolved onto SDS-polyacrylamide gels (Beyotime Biotechnology) and blotted onto PVDF membranes (Hybond P, GE Healthcare Bio-Sciences, Pittsburgh, Pennsylvania). Primary antibodies against phosphorylated and total STAT3 (#4093s, #12640s, Cell Signaling Technology), anti-ATF4 (#11815, cell signaling), anti-caspase 3 (#9579, cell signaling), anti-LC3II/I (#4108, cell signaling), anti-P62 (#5114s, cell signaling), anti-ATG7 (#2631, cell signaling) and GADPH (#2118, cell signaling) were used. Images were performed using a C-Digit chemiluminescent Western blot scanner (LI-COR, Lincoln, USA).

### Quantitative real-time RT-PCR

TRIzol reagent was used for total RNA extraction (Invitrogen Life Technologies, Carlsbad, CA, USA) according to the manufacturer’s instructions. Total RNA was reversely transcribed with random primer and M-MLV Reverse Transcriptase (Takara). Quantitative real-time RT-PCR was performing using SYBR Green I Master Mix reagent by ABI 7500 system (Applied Biosystem, Foster, CA, USA). Primers used in this study is shown in Supplementary Table 1. Results were calculated by using 2^−(ΔΔCt)^ method.

### Statistical analysis

Data that were not normally distributed were compared with Mann–Whitney *U* test and expressed as median (interquartile range, IQR), and data with a normal distribution were compared with Student’s *t* test and presented as means ± standard error (SE). Serum IL-22 levels in pediatric patients were expressed as scatter dot plots with medians. To determine the discriminative power of IL-22 for SALI, receiver operating characteristic (ROC) curves were constructed and the area under the ROC curve (AUC) was determined with its 95% confidence interval (*CI*). Serum IL-22 levels in mice were expressed as scatter dot plot with medians. All figures were done using GraphPad Prism version 6.0 (Graph-Pad Software, San Diego, CA). Significant differences were analyzed by Student’s *t* test or Mann–Whitney *U* test using STATA 15.0 MP (College Station, Texas, USA). Values of *p* < 0.05 were considered statistically significant.

## Results

### Serum IL-22 levels in pediatric patients with sepsis or LPS-treated mice

**Fig. 1:**
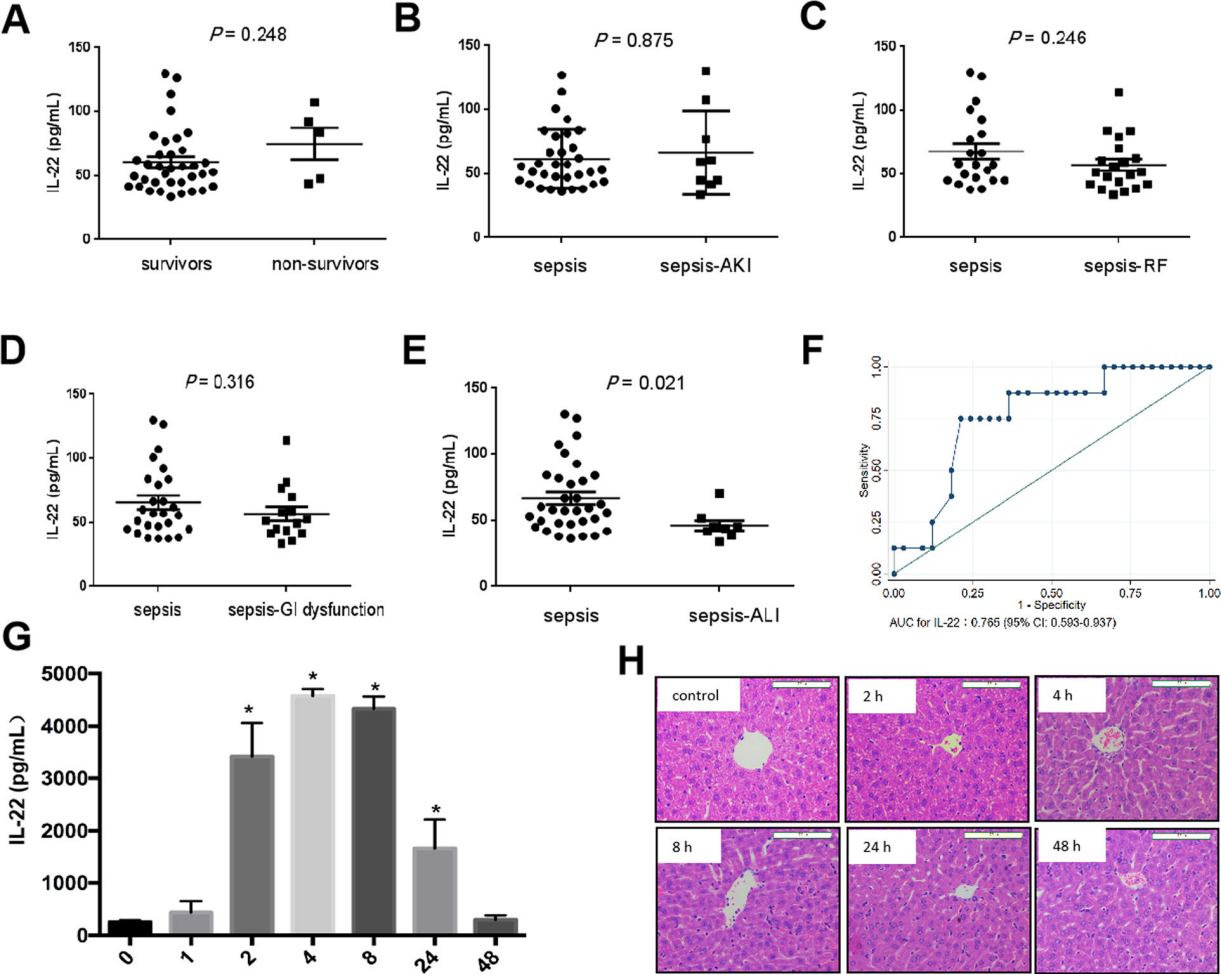
Serum IL-22 levels on PICU admission were decreased in septic patients with liver injury and the fall-down of serum IL-22 level was associated with LPS-induced acute liver injury. IL-22 concentrations were measured by ELISA in serum samples collected from 41 patients with sepsis (sepsis). **a** Serum IL-22 concentrations in survivors and non-survivors with sepsis, **b** serum IL-22 concentrations in septic patients with (sepsis-AKI) or without acute kidney injury (sepsis), **c** serum IL-22 concentrations in septic patients with (sepsis-RF) or without respiratory failure (sepsis), **d** serum IL-22 concentrations in septic patients with (sepsis-GI dysfunction) or without gastrointestinal (GI) dysfunction (sepsis), **e** serum IL-22 concentrations in septic patients with (sepsis-ALI) or without acute liver injury (sepsis), **f** ROC curve for IL-22 as a predictor for sepsis-associated liver injury (SALI), **g** serum IL-22 concentrations in LPS-treated mice at indicated time (1, 2, 4, 8, 24, 48 h) after LPS treatment (5 mg/kg body weight, i.p, once) (*n* = 5). **h** Hematoxylin and Eeosin staining (*H&E* staining) of liver sections from mice treated with LPS for different time. ^*^ indicates the significant difference compared with control group (0 h), *p* < 0.05.

The baseline characteristics of pediatric patients with sepsis were presented in Supplementary Table 2. The PICU mortality was 12.2% (5/41) in these septic patients. There were no differences in serum IL-22 levels between non-survivors and survivors (Fig. 1a) and in different subgroups of sepsis-AKI, sepsis-RF, sepsis-GI dysfunction (Fig. 1b–d). However, serum IL-22 concentrations on PICU admission were significantly lower in patients with SALI compared to septic patients without liver injury (*p* = 0.021, Fig. 1e). Multivariate *logistic* analysis indicated that IL-22 was not independently associated with SALI adjusted by PRISM III and age (OR: 0.922 [95% CI: 0.842–1.009]) (Supplementary Table 3). Further analysis indicated that the AUC for IL-22 discriminating SALI was 0.765 (95% CI: 0.593–0.937) (Fig. 1f).

In LPS-treated mice, serum levels of IL-22 increased gradually from 1 h and peaked at 4 h after LPS treatment. The high levels of IL-22 maintained until to 8 h after LPS treatment, and then decreased to normal level at 48 h after LPS treatment (Fig. 1g). Compared with control group, liver sections with centrilobular necrosis, inflammatory infiltration and ductal proliferation were aggravated at 24 h and 48 h after LPS treatment (Fig. 1h).

### Pre-treatment with recombinant murine IL-22 protects mice against LPS-induced acute liver injury

**Fig. 2:**
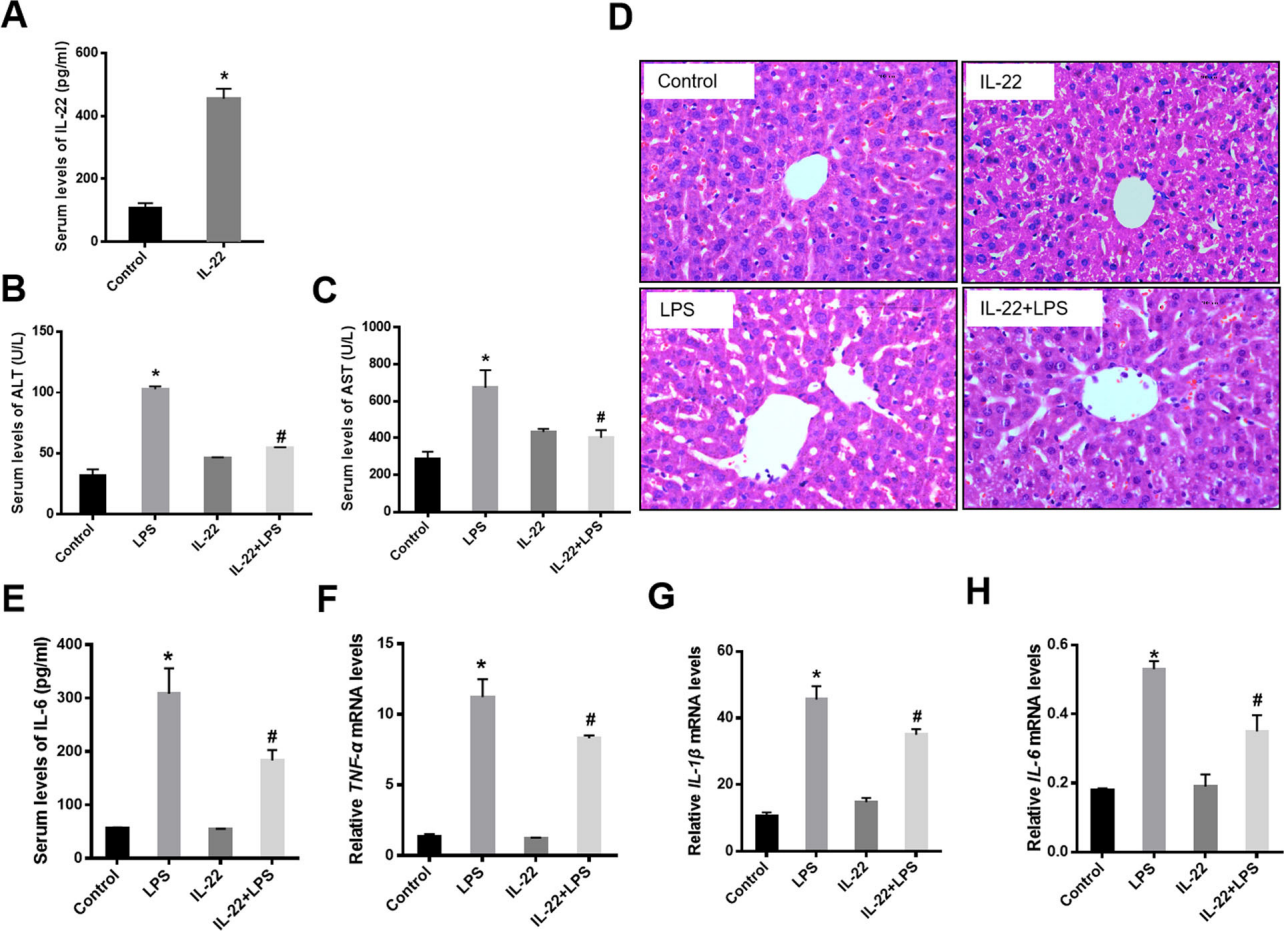
IL-22 protects mice against LPS-induced acute liver injury. Mice were divided into control (Control), LPS-treated (LPS), IL-22 only (IL-22), and IL-22 pretreatment for 4 days before LPS administration (IL-22+LPS) groups (*n* = 6). **a** Serum IL-22 levels in mice with (IL-22) or without IL-22 pre-treatment (Control). **b** Serum levels of ALT. **c** Serum levels of AST. **d** Liver sections were stained with hematoxylin and eosin (*H&E* staining) at 48 h after LPS stimuli. **e** Serum levels of IL-6. **f** The mRNA levels of TNF-α in livers. **g** The mRNA levels of IL-1β in livers. **h** The mRNA levels of IL-6 in livers. ^*^ indicates the significant difference compared with Control group, *p* < 0.05. ^*#*^ indicates the significant difference compared with LPS group, *p* < 0.05.

To explore the role of IL-22 in SALI, mice were pre-treated the recombinant murine IL-22 for 4 days before LPS administration. In the group of IL-22 pre-treatment, serum IL-22 levels were higher than that in the control group (Fig. 2a). In response to LPS stimulation, the elevated levels of ALT and AST in response to LPS stimulation were ameliorated in IL-22 pre-treated mice compared to LPS-challenged mice (Fig. 2b, c). Consistently, histopathology demonstrated that there were no obvious inflammatory infiltration and centrilobular necrosis in the livers of IL-22 pre-treated mice (Fig. 2d). In addition, serum IL-6 levels were significantly lower in mice with IL-22 pre-treated compared with LPS-treated mice (Fig. 2e). Moreover, the mRNA levels of TNF-α, IL-1β, and IL-6 in livers were significantly suppressed in mice with IL-22 pre-treatment (Fig. 2f–h).

### IL-22 administration activates hepatic autophagy in vitro and in vivo

**Fig. 3:**
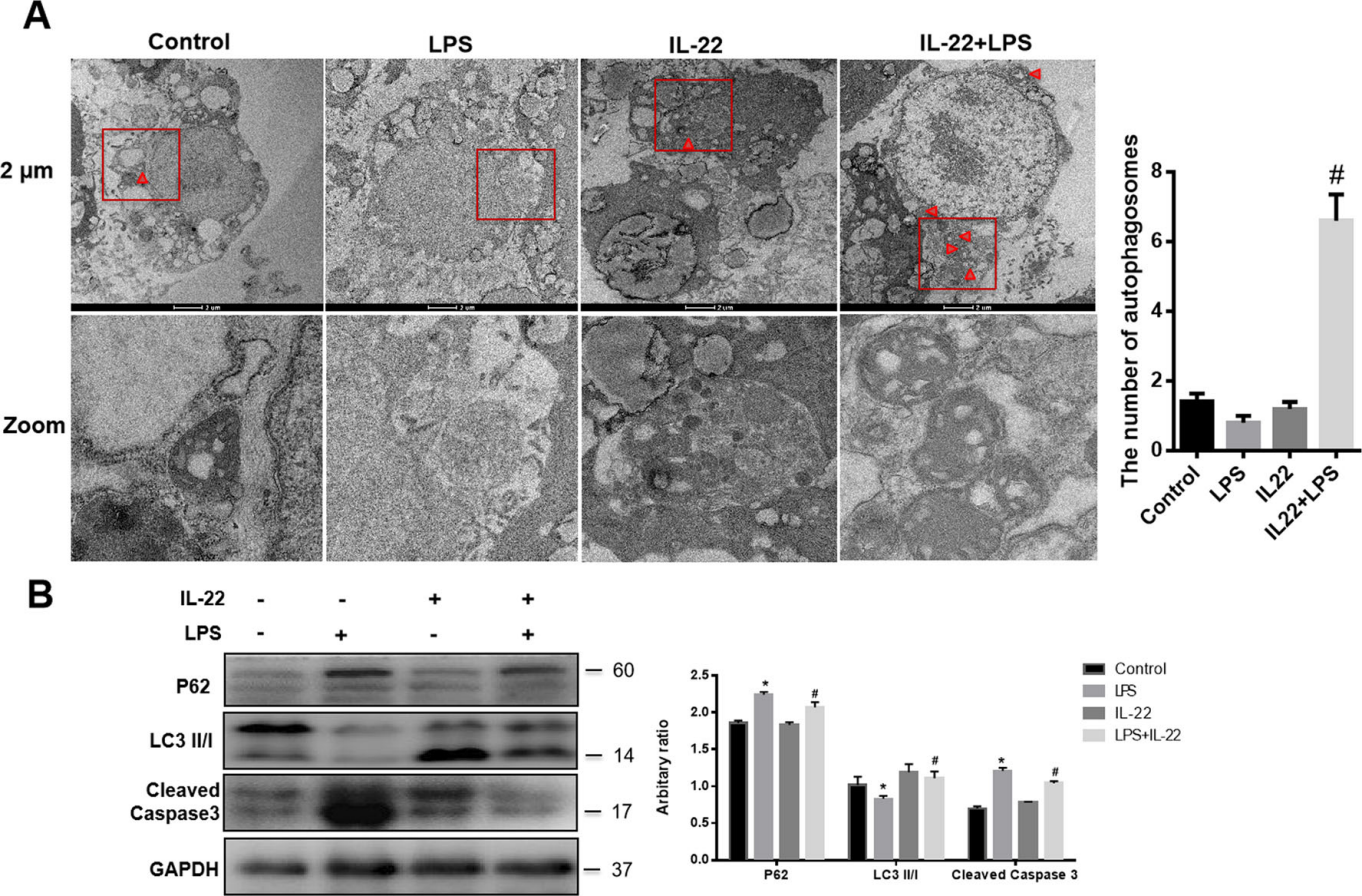
IL-22 administration activates autophagy in livers. **a** HepG2 cells were processed for transmission electron microscopy (*n* = 3) (left) with quantitative analysis (right). Red arrows indicate the number and location of autophagosomes. **b** The protein levels of P62, LC3II/I, and Cleaved Caspase 3 in livers of mice (left) and the quantity of the protein levels (right). * indicates the significant difference compared with Control group, *p* < 0.05, ^*#*^ indicates the significant difference compared with LPS group, *p* < 0.05.

Sepsis-induced autophagy occurs transiently in hepatocytes at early stage and then is suppressed at later phase^19^. Recent studies indicated that autophagy activation prevents hepatocyte apoptosis and improves functional recovery of liver failure during sepsis^20,21^. To investigate the potential role of IL-22 in regulating autophagy in hepatocytes, results obtained from transmission electron microscopy showed that the number of autophagosomes in HepG2 cells was decreased upon LPS challenge for 8 h, and there were more autophagosomes in HepG2 cells with IL-22 pre-treatment for 24 h (Fig. 3a). Furthermore, LPS suppressed the expression of LC3II/I and increased the expression of P62, which were reversed in livers by IL-22 pre-treatment. Cleaved Caspase 3 is used as a biomarker of apoptosis^22^. Activation of autophagy can alleviate and prevent apoptosis^23^. The levels of Cleaved Caspase 3 were decreased in livers of mice pre-treated with IL-22 for 4 days (Fig. 3b).

### IL-22 attenuates LPS-induced cytotoxicity via autophagy activation in vitro

**Fig. 4:**
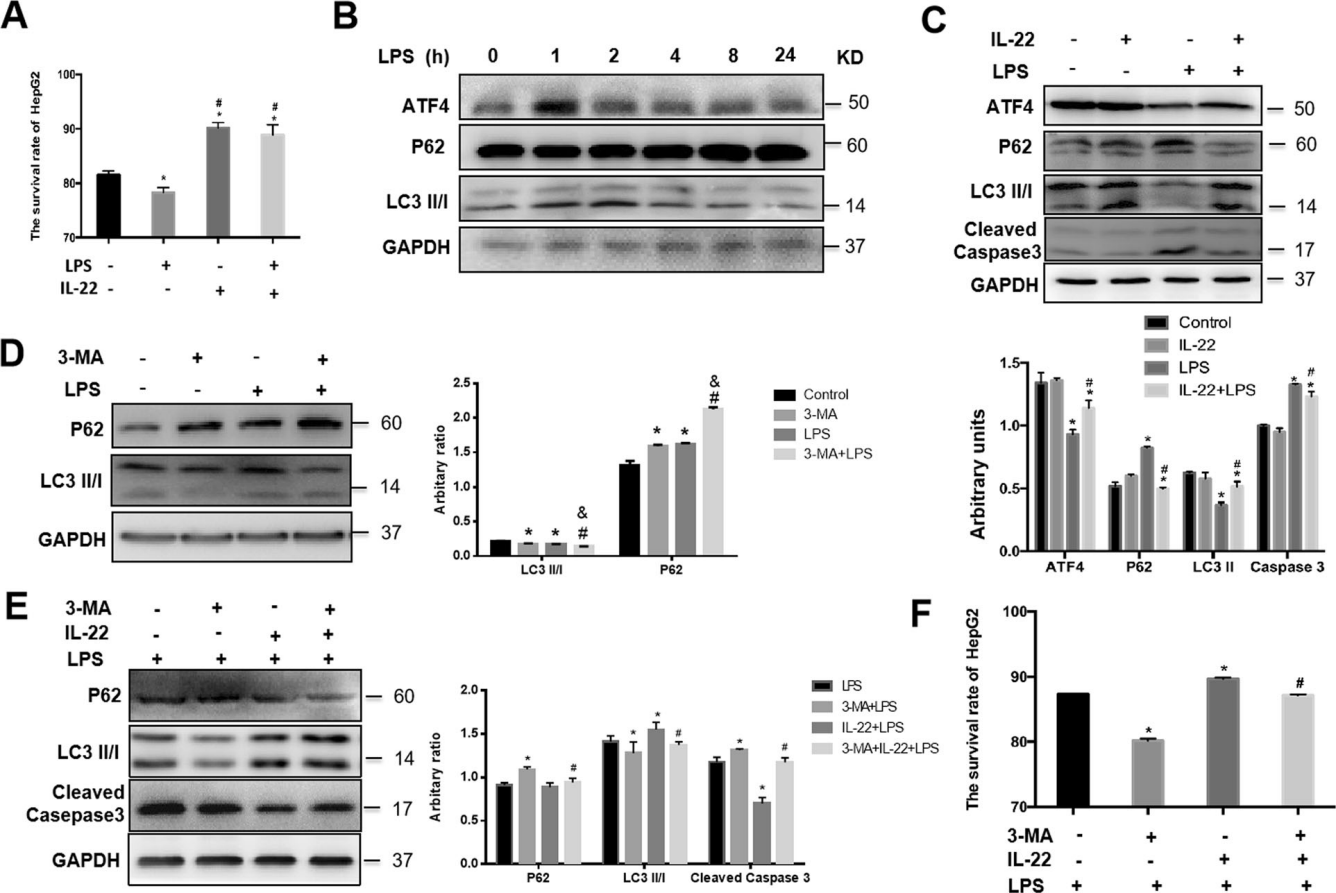
IL-22 attenuates LPS-induced cytotoxicity *via* autophagy activation in HepG2 cells. **a** The survival rate of HepG2 cells with or without IL-22 pretreatment in response to LPS stimuli. **b** The protein level of ATF4, P62, LC3II/I in HepG2 cells after LPS treated at indicated time. **c** The protein levels of ATF4, P62, LC3II/I, and Cleaved Caspase 3 in HepG2 cells (upper) and the quantity of the protein levels (lower). **d** The protein levels of P62 and LC3II/I in HepG2 cells treated with 3-MA (left) and the quantity of the protein levels (right). **e** The protein levels of P62, LC3II/I, and Cleaved Caspase 3 in HepG2 cells with IL-22 pre-treatment, then 3-MA blocking autophagy followed by LPS treatment (left) and the quantity of the protein levels (right). **f** The survival rate of HepG2 cells in response to IL-22 pre-treatment with or without 3-MA treatment followed by LPS stimulation. * indicates the significant difference compared with Control group (**a** and **c:** without LPS or IL-22 group, **d**: without 3-MA or LPS, **e**: LPS group), *p* < 0.05. ^#^ indicates the significant difference compared with LPS group (**a** and **c**) or 3-MA group (**d**) or 3-MA + LPS (**e** and **f**), *p* < 0.05, ^&^ indicates the significant difference compared with LPS group (**d**).

To further investigate the role of autophagy activation in the process of LPS-induced hepatocyte apopotosis, HepG2 cells were pre-treated with IL-22 (10 ng/ml) for 24 h, then were treated with LPS for another 8 h. LPS challenge significantly decreased cell viability in HepG2 cells and it was restored by IL-22 pre-treatment (Fig. 4a). Considering that ATF4 is an upstream regulator of autophagy^24,25^, the expression of ATF4 protein was determined. Under the condition of LPS administration, the expression of ATF4 in HepG2 cells was increased rapidly and peaked at 1 h after LPS stimulation and the expression of LC3II peaked at 2 h after LPS stimulation (Fig. 4b). Both ATF4 and LC3II reduced at 4 h after LPS treatment (Fig. 4b). The expression of P62 was increased at 8 h after LPS treatment (Fig. 4b). At 8 h after LPS treatment, IL-22 pre-treatment stimulated the expression of ATF4 and LC3II, but suppressed the expression of P62 and Cleaved Caspase 3 (Fig. 4c).

To confirm the potential roles of autophagy activation in mediating IL-22 protective effects, 3-methyladenine (3-MA) was used to inhibit autophagy in HepG2 cell lines. LC3II induction was suppressed in the presence of 3-MA in response to LPS treatment (Fig. 4d, e). Suppression of autophagy by 3-MA promotes the apoptosis of HepG2 cells indicated by the increasing levels of Cleaved Caspsase 3 (Fig. 4e). Moreover, cell viability was significantly decreased after treated with 3-MA (Fig. 4f).

### ATF4-ATG7 signaling pathway mediates autophagy activation in response to IL-22 administration

**Fig. 5:**
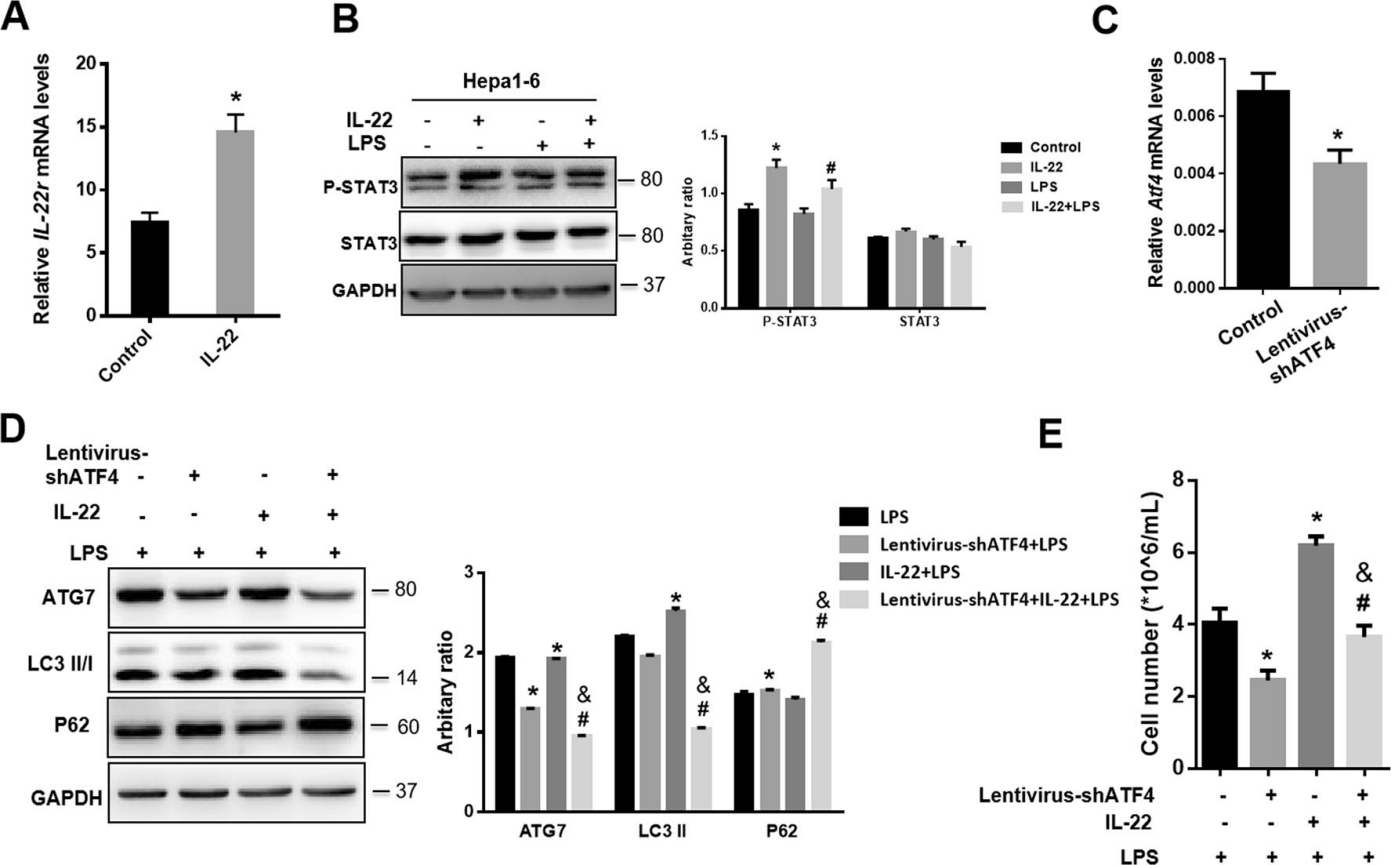
ATF4-ATG7 signaling pathway mediates autophagy activation in response to IL-22 administration. **a** The mRNA levels of IL22r in livers of mice with (IL-22) or without IL-22 pre-treatment (Control). **b** The expression of P-STAT3 and STAT3 in Hepa1-6 cells (Control: without IL-22 pre-treatment without LPS stimulation; IL-22: IL-22 pre-treatment only; LPS: without IL-22 pre-treatment and with LPS stimulation; IL-22+LPS: with IL-22 pre-treatment followed by LPS stimulation). **c** The mRNA levels of ATF4 (Control: lentivirus-control; Lentivirus-shATF4: ATF4 siRNA knockdown). **d** The protein levels of ATG7, LC3II/I, and P62 in Hepa1-6 cells in response to LPS treatment (right) and the quantity of the protein levels (left) (LPS: only with LPS stimuli; Lentivirus-shATF4+LPS: ATF4 siRNA with LPS stimulation; IL-22+LPS: with IL-22 pre-treatment followed by LPS stimuli; Lentivirus-shATF4+IL-22+LPS: after ATF4 knockdown then IL-22 pre-treatment followed by LPS stimuli). **e** Cell counts of Hepa1-6 cells in response to IL-22 pre-treatment with or without ATF4 knockdown followed by LPS stimuli. ^*^indicates the significant difference compared with Control group (**a** and **c**) or group without IL-22 or Lentivirus-shATF4 treatment (**d** and **e**), *p* < 0.05. ^#^indicates the significant difference compared with LPS group (**b**) or Lentivirus-shATF4+LPS group (**d** and **e**), *p* < 0.05. ^&^indicates the significant difference compared with IL-22+LPS group (**d** and **e**), *p* < 0.05.

IL-22 binds to IL-22R and then stimulates STAT3 signaling^9^. The IL-22R mRNA levels were significantly upregulated in livers of mice treated with recombinant murine IL-22 than those in controls (Fig. 5a). Further analysis indicated that the levels of phosphorylated STAT3 (P-STAT3) was significantly increased in response to IL-22 administration, but there was no difference between with or without LPS stimulation in Hepa1–6 cells (Fig. 5b).

Considering that ATF4 is an upstream regulator of autophagy^24,25^ and the expression of ATF4 protein was regulated by LPS and IL-22 in cells (Fig. 4b, c), we hypothesized that IL-22 could stimulate ATF4 expression, and then activate autophagy. To test this possibility, ATF4 was knocked down *via* lentivirus-mediated shATF4, and the expression of *Atf4* mRNA was significantly suppressed in Hepa1-6 cells (Fig. 5c). As a result, the expression of ATG7, as a direct target gene of ATF4, was significantly decreased in the group of ATF4 knockdown, and the enhancement of LC3II formation by IL-22 pre-treatment was abolished by ATF4 knockdown (Fig. 5d). Moreover, the reduction of ATF4 promoted the protein levels of P62 (Fig. 5d). Consequently, the effects of IL-22 protecting cell apoptosis against LPS were blocked. According to the results, cell number of Hepa1-6 was significantly lower when ATF4 was knocked down compared with control group (Fig. 5e).

### ATF4 knockdown aggravates LPS-induced acute liver injury in mice associated with ATG7-mediated autophagy suppression

**Fig. 6:**
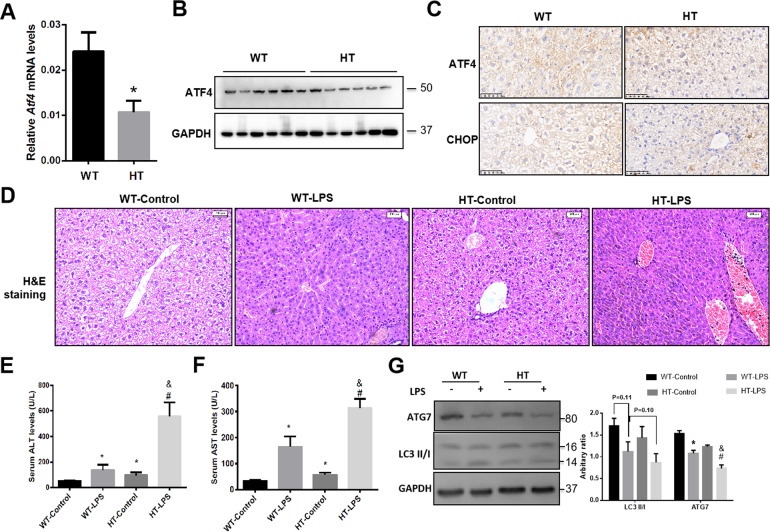
ATF4 knockdown aggravates LPS-induced acute liver injury in ATF4 knockdown (HT) mice. **a**–**g** ATF4 knockdown (HT) mice and wild-type (WT) mice were treated with LPS (5 mg/kg) for 24 h (*n* = 6). **a** *Atf4* mRNA levels in livers, **b** ATF4 protein levels in livers, **c** immunochemistry of ATF4 and CHOP expression in livers, **d** H&E staining of liver section, **e** serum ALT levels, **f** serum AST levels, **g** the protein levels of LC3II/I and ATG7 (right) and the quantity of the protein levels (left). * indicates the significant difference compared with WT (**a**) or WT-Control group (**e, f** and **g**), ^#^ indicates the significant difference compared with WT-LPS group (**e, f** and **g**), *p* < 0.05. ^&^ indicates the significant difference compared with HT-control group (**e, f** and **g**).

To further investigate the role of ATF4 in mediating the protective role of IL-22 in LPS-induced ALI, *Atf4*^*+/*−^ mice were used. The mRNA and protein levels of ATF4 were decreased to about 50% in livers of *Atf4*^*+/*−^ mice (Fig. 6a, b). The results of immunohistochemistry showed that the expression of ATF4 and its downstream target CHOP was decreased in livers of *Atf4*^*+/*−^ mice (Fig. 6c). In response to LPS stimulation, histopathology demonstrated that inflammatory infiltration and centrilobular necrosis in livers of *Atf4*^*+/*−^ mice were significantly worse than that in wild-type mice (Fig. 6d). In addition, the elevated levels of ALT and AST were aggravated in *Atf4*^*+/*−^ mice compared with wild-type mice (Fig. 6e, f). Moreover, the expression of LCII and ATG7 was suppressed in livers of *Atf4*^*+/*−^ mice in response to LPS comparing with wild-type mice (Fig. 6g).

### The protective effects of IL-22 on LPS-induced acute liver injury is partially blocked in ATF4 knockdown (*Atf4*^*+/*−^) mice

**Fig. 7:**
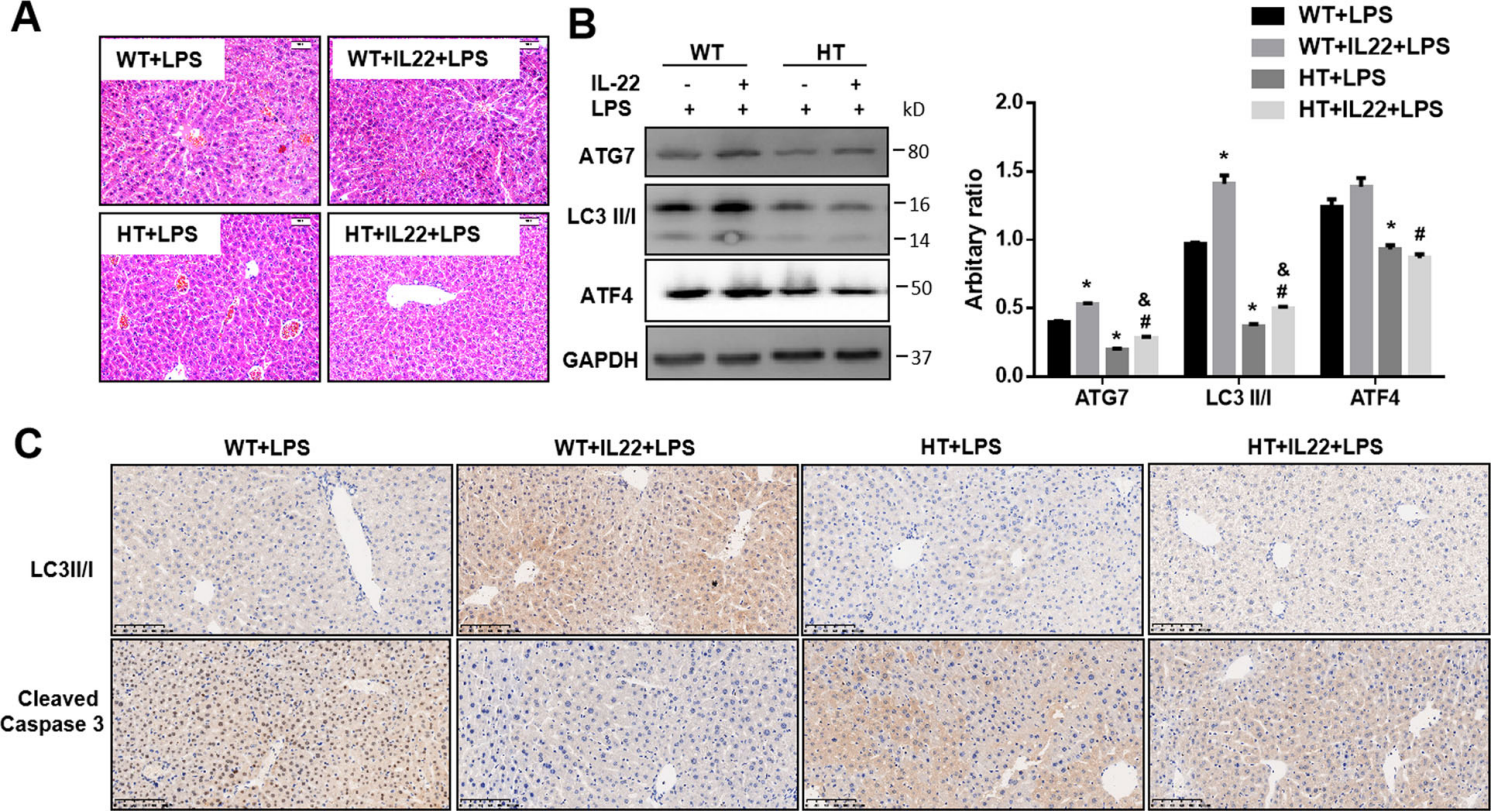
The protective effects of IL-22 on LPS-induced acute liver injury is partially blocked in ATF4 knockdown (HT) mice compared with wild-type (WT) mice. **a**–**c** ATF4 knockdown (HT) mice and wild-type (WT) mice were pretreated with or without recombinant murine IL-22 (IL-22) for 4 days, followed by LPS treatment (LPS) for 24 h (*n* = 4). **a** H&E staining of liver section, **b** the protein levels of ATG7, LC3II/I and ATF4 (right) and the quantity of the protein levels (left), **c** immunochemistry of LC3II/I and Cleaved Caspase 3 expression in livers of mice. ^*^ indicates the significant difference compared with WT + LPS group. ^#^ indicates the significant difference compared with WT + IL-22+LPS group, *p* < 0.05. ^&^ indicates the significant difference compared with HT + LPS group.

Using *Atf4*^*+/*−^ mice, we tried to assess the importance of ATF4 in mediating the effects of IL-22 on LPS-induced ALI. The results showed that the inflammatory infiltration and bleeding in livers of *Atf4*^*+/*−^ mice were worse than that in wild-type mice in response to LPS compared with wild-type mice, and the protective effects of IL-22 was partially blocked in *Atf4*^*+/*−^ mice (Fig. 7a). The effects of IL-22 on stimulating the expression of ATG7 and LC3II in livers were blocked by ATF4 knockdown (Fig. 7b). In addition, the expression of LC3II/I in livers of mice with pre-treatment of IL-22 was increased in wild-type mice accompanied with low expression of Cleaved Caspase 3, which were suppressed in livers of *Atf4*^*+/*−^ mice pre-treated with IL-22 (Fig. 7c).

## Discussion

**Fig. 8:**
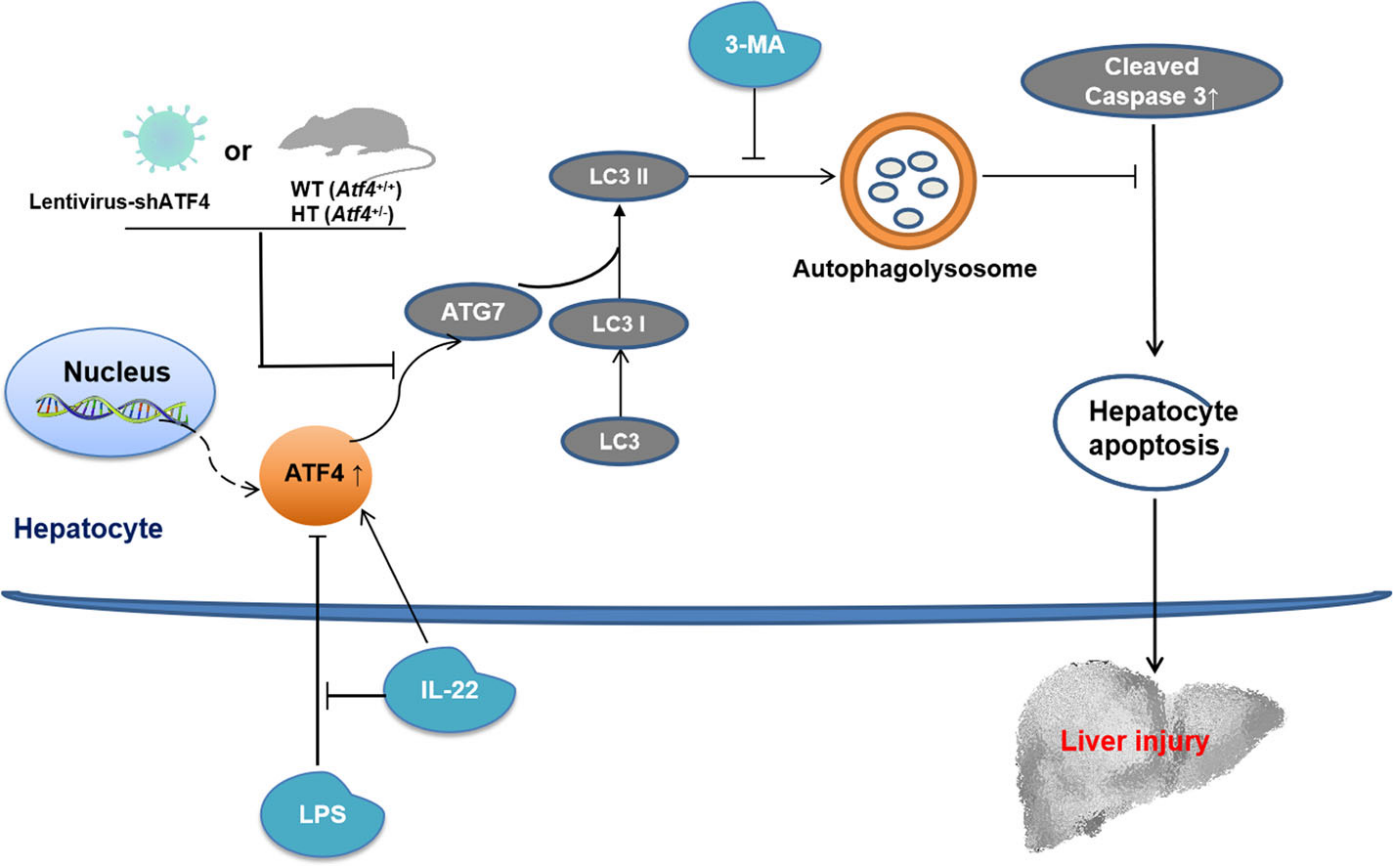
Working model about IL-22 protecting mice against LPS-induced acute liver injury. IL-22 activates autophagy in hepatocytes via stimulating ATF4-ATG7 signaling pathway and protects hepatocytes against LPS-induced apoptosis. Pre-treatment of IL-22 is effective to alleviate LPS-induced acute liver injury.

In the present study, we revealed that serum IL-22 levels were lower in pediatric SALI than those without liver injury, and pre-treatment with recombinant murine IL-22 protects mice against LPS-induced ALI. Further study enclosed that IL-22 activates autophagy in vivo and in vitro and plays an important role against LPS-induced ALI, which is partially mediated via stimulating the ATF4-ATG7 signaling pathway (Fig. 8).

To our best knowledge, we firstly reported the serum levels of IL-22 in pediatric patients with sepsis, and found that serum IL-22 levels were significantly lower in patients with SALI than those in patients without liver injury, but the multivariate logistic analysis showed that IL-22 was not independently associated with SALI after adjusted by PRISM III and age (*p* = 0.077). We suspected that the small sample size might affect the power of statistic analysis. The specific association between IL-22 and SALI needs further investigation in a larger population. In terms of molecular mechanisms, the high expression levels of IL-22R1 in liver progenitor cells and hepatic stellate cells make IL-22 an attractive agent for study in chronic liver disease^26^. Given that hepatocytes possess a large repertoire of IL-22 receptor complex, IL-22 plays a hepato-protective role during liver disease^14–16^. So, we suspected that the abundant expression of IL-22R1 in livers could be main cause of the specific roles of IL-22 in SALI.

It is well known that SALI mainly develops in the stepwise process of overwhelming inflammatory response^2^. In the present study, our data demonstrated that pretreatment with IL-22 diminished IL-6 levels in serum and livers. Emerging evidence showed that STAT3-mediated signaling is a common pathway utilized by IL-22 in cell proliferation and regeneration under inflammation status^8^. In vivo pretreatment of IL-22 is beneficial in acetaminophen-induced hepatotoxicity partially due to the activation of STAT3 signals^22^. Furthermore, STAT3 signaling is believed to promote lung cancer cell proliferation and migration and stimulate hepatocyte compensatory proliferation^23,27^. The expression of P-STAT3 was stimulated in response to IL-22 pre-treatment, either with or without LPS stimuli. These results suggested that IL-22 protects hepatocytes from LPS-induced damage in a STAT3-dependent manner.

In this study, we found that IL-22 induces the expression of ATF4 in hepatocytes and livers. Previous study indicated that the expression of ATF4 was decreased in inflamed intestinal mucosa in patients with active inflammatory bowel diseases (IBD), and overexpression of ATF4 could down-regulated inflammatory factors including IL-1β and IL-6, deprivation of ATF4 promotes intestinal inflammation^28^. Recently, ATF4 has been reported to have protective properties upon neurological damages by ameliorated inflammation^29^. Therefore, ATF4 might be a regulator involved in inflammation response during sepsis. The suppressive effects of IL-22-ATF4 pathway on LPS-induced inflammatory response should be paid more attention as a target for reducing inflammatory damage.

Autophagy regulates innate immune response and plays a negative role in inflammation activation^30,31^. Autophagy occasionally is a crucial process of pathogenesis in some diseases. When treated with lysosomal inhibitors, the level of LC3II in acinar cells of pancreatitis models was higher than control’s^32^. Liver-specific IL-22 transgenic mice or pre-treatment wild type mice with IL-22 were significantly resistant to cerulein-induced pancreatitis by inhibiting autophagic pathway^33^. Inhibition of autophagosome completion may contribute to the exacerbation in the development of acute pancreatitis^34^. During sepsis, autophagy prevents apoptosis and improves functional recovery of liver failure^20,21^. Moreover, suppression of autophagy promotes the aberrant upregulation of apoptosis and aggravates hepatocytes dysfunction^35^. Liver-specific deletion of the autophagy gene promotes hepatomegaly, fibrosis, benign adenomas and hepatocarcinogenesis. All these above findings support that the functional autophagy exerts protective effect on liver injury during sepsis. In our study, serum IL-22 levels increased at 2 h and remained high levels until 8 h after LPS treatment, then gradually declined within 48 h companied with aggravating liver damage. Consistently, autophagy occurs transiently in hepatocytes at the early stage and then is suppressed at the later phase^19^. In the present study, IL-22 activates autophagy in livers of mice treated by LPS, and suppression of autophagy by 3-MA exerts detrimental effects on LPS-treated cells. To our knowledge, this is the first report about the role of IL-22 in regulation of autophagy activation in hepatocytes during sepsis. The elevated level of IL-22 in early stage may protect liver against damage, possibly related to autophagy activation. In another respect, IL-22 promotes proliferation and survival of human HepG2 cells by inducing expression of a large amount of anti-apoptotic and mitogenic proteins^14^. Consistently, the expression of Cleaved Caspase 3 in livers or in HepG2 cells confirmed the potential roles of IL-22 in anti-apoptosis. Whether there is correlation between IL-22-mediated autophagy activation and inducing anti-apoptotic protein needs further investigation.

In this study, we proved that ATF4-mediated autophagy activation plays a crucial role in IL-22 protecting hepatocytes against LPS-induced damage. Previous investigations described that intermittent hypoxia induced autophagy activation is dependent on PERK/eIF2α/ATF4 signaling pathway, inhibition of autophagy by chloroquine or deficiency of autophagy-related gene 5 (Atg5) and Atg7 promoted pancreatic β-cell apoptosis^36^. In addition, ATF4 depletion promotes hepatocytes apoptosis by inhibiting autophagy^36,37^. Collectively, our study suggests that ATF4 is an essential downstream molecule of IL-22-activated autophagy in hepatocytes. Thus, ATF4 fulfills crucial functions of activating autophagy to exert protective properties. Previous study indicated that IL-22 enhanced AMPK-dependent autophagy in acetaminophen-induced liver injury^12^. Whether AMPK signaling pathway mediates ATF4-related autophagy activation needs further study.

Several limitations of our study need consideration. Firstly, this is a pilot study to explore the potential roles of IL-22 in SALI, and the small sample size of patients with sepsis affected the assessment of IL-22 for discriminating SALI from sepsis, and this conclusion requires further investigation in a larger population. Secondly, considering that the microenvironment is different between normal cells and liver cancer cell lines, mouse primary cells should be used to confirm the results obtained from HepG2 and Hepa1-6. Thirdly, LPS-induced ALI could be limited to mimic SALI, and further study should consider the model of cecal ligation and perforation in mice. Fourthly, cytokine and molecular networks may be further used to analysis two or many factors interaction^38^. Although the capacity of IL-22 for discriminating SALI showed an AUC of 0.765 (95% CI: 0.593, 0.937), the combination of IL-22 with other indexes could enhance the accuracy for early recognition of SALI in pediatric patients. In addition, measured levels of IL-22 at different days after admission in children with sepsis, or in children with non-septic liver injury or healthy children were unavailable due to the lack of blood samples in children in the present study. Fifthly, due to low fertility of ATF4 knockout (*Atf4*^−*/*−^) mice, *Atf4*^*+/*−^ mice was used in this study to mimic the reduced expression of ATF4. At last, the kinetics of IL-22 secretion and the factors regulating IL-22 production in patients with sepsis remain unknown and should be investigated in the future.

In conclusion, it is the first report about the association of serum IL-22 with SALI and the protective effects of IL-22 pre-treatment on LPS-induced ALI. IL-22 activates autophagy in hepatocytes *via* ATF4-ATG7 signaling pathway. These findings give new insights into IL-22-ATF4 mediated autophagy activation as a potential therapeutic strategy to SALI.

## Supplementary information

Supplementary Table 1

Supplementary Table 2

Supplementary Table 3

Datasheet for serum IL-22 in children with sepsis

## Data Availability

The datasheet for determining serum IL-22 levels in children with sepsis was submitted as a supplementary material, and the file has been removed the private information about patients.
