# Supplementary material for: IL-22 ameliorates LPS-induced acute liver injury by autophagy activation through ATF4-ATG7 signaling
Source: Cell Death Dis. 2020 Nov 11;11(11):970. doi: 10.1038/s41419-020-03176-4 (PMC7658242; doi:10.1038/s41419-020-03176-4)
Supplement: Supplementary file 1 — Supplementary Table 1 [file 41419_2020_3176_MOESM1_ESM.docx]

**Supplementary Table 1. Primers used in this study**

| **Gene** | **Forward primer (5’-3’)** | **Reverse primer (5’-3’)** |
| --- | --- | --- |
| *Il-22r* | TCTCCATCCTGCAACCTACC | TGTCTTCCTGGAGCTGACCT |
| *Il-1β* | TCGTGCTGTCGGACCCATAT | GTGTGCCGTCTTTCATTACA |
| *Tnf-α* | ATGAGCACAGAAAGCATGATC | TACAGGCTTGTCACTCGAATT |
| *Il-6* | TGTCTATACCACTTCACAAGTCGGAG | GCACAACTCTTTTCTCATTTCCAC |
| *Gadph* | AGGTCGGTGTGAACGGATTTG | GGGGTCGTTGATGGCAACA |
