# Supplementary material for: IL-22 ameliorates LPS-induced acute liver injury by autophagy activation through ATF4-ATG7 signaling
Source: Cell Death Dis. 2020 Nov 11;11(11):970. doi: 10.1038/s41419-020-03176-4 (PMC7658242; doi:10.1038/s41419-020-03176-4)
Supplement: Supplementary file 2 — Supplementary Table 2 [file 41419_2020_3176_MOESM2_ESM.docx]

**Supplementary Table 2.** **Baseline characteristics and outcome of patients with sepsis**

| **Characteristics** | **Total (n = 41)** | **Sepsis (n =33 )** | **Sepsis-liver injury (n =8 )** | ***P*** |
| --- | --- | --- | --- | --- |
| Age (month) | 19 (9.5, 60) | 19 (10, 60) | 17.5 (6.8, 50) | 0.895 |
| PRISM III | 7 (3, 10) | 6 (3, 10) | 9.5 (7.5, 11) | 0.415 |
| WBC (×10^9^/L) | 10.2 (6.8, 14.9) | 10.9 (7, 15.1) | 7.7 (3.9, 13.4) | 0.278 |
| CRP (mg/L) | 74 (6, 120) | 67 (5, 111) | 104 (14, 170) | 0.134 |
| IL-22 (pg/mL) | 55.5 (44.8, 76.8) | 57.7 (47.7, 81.3) | 44.3 (39.9, 48.1) | 0.021 |
| ALT (U/L) | 19 (13, 48) | 17 (13, 28) | 69.5 (50.5,185) | 0.004 |
| AST (U/L) | 38 (27,55) | 35 (27,42) | 217 (66.5, 317.5) | 0.003 |
| r-GT | 17 (10, 27) | 13 (9, 27) | 23 (17.5, 111.5) | 0.078 |
| TBIL | 8.3 (5.7, 12.5) | 7.6 (5.3, 12.5) | 8.5 (7.4, 24.6) | 0.576 |
| DBIL | 2.7 (1.6, 5.8) | 2.7 (1.6, 5.8) | 3.1 (1.3, 21.5) | 0.869 |
| WBC | 10.2 (6.8, 14.9) | 10.9 (7, 15.1) | 7.7 (3.9, 13.4) | 0.278 |
| PLT | 255 (156, 308) | 272 (156, 326) | 176 (142, 234.5) | 0.052 |
| BUN | 3.5 (2.6, 5.4) | 3.5 (2.7, 5.3) | 3.9 (2.1, 9.5) | 0.742 |
| Length of PICU stay, day | 7 (5, 12) | 6 (4, 10) | 11 (7.5, 36) | 0.012 |
| PICU mortality, n (%) | 5 (12.2) | 4 (12,1) | 1 (12.5) | 0.977 |

PRISM III: pediatric risk of mortality III；WBC: white blood cell; CRP: C-reactive protein; IL-22: Interleukin-22; ALT: alanine aminotransferase; AST: aspartate transaminase; γ-GT:γ-glutamyl transferase ; TBIL: total bilirubin; DBIL: direct bilirubin; WBC: white blood cell; PLT: platelet; BUN: blood urea nitrogen; ALB:[albumin](C:/Program%20Files%20(x86)/Youdao/Dict/8.9.3.0/resultui/html/index.html#/javascript:;); PICU: pediatric intensive care unit.
