# Supplementary material for: IL-22 ameliorates LPS-induced acute liver injury by autophagy activation through ATF4-ATG7 signaling
Source: Cell Death Dis. 2020 Nov 11;11(11):970. doi: 10.1038/s41419-020-03176-4 (PMC7658242; doi:10.1038/s41419-020-03176-4)
Supplement: Supplementary file 3 — Supplementary Table 3 [file 41419_2020_3176_MOESM3_ESM.doc]

**Supplementary Table 3.** Multivariate logistic analysis of the association of IL-22 with sepsis-associated liver injury adjusted by PRISM III score and age

| **Variables** | ***OR*** | **95% *CI*** | ***P*** |
| --- | --- | --- | --- |
| IL-22 | 0.922 | 0.842-1.009 | 0.077 |
| PRISM III | 1.039 | 0.813-1.328 | 0.758 |
| Age | 1.008 | 0.983-1.033 | 0.542 |
